# Most Serious Objection

**Published:** 1910-06-04

**Authors:** 


### "MOST SERIOUS OBJECTION."

Sir,—We have read with interest the Special Department article in your issue of May 7, on p. 177, and take the most serious objection to the prices given for surgical dressings, as scarcely in any single instance could dressings of even an inferior quality be obtained at the figures, and in many instances the articles that could be bought would have to be of a very inferior and poor quality; in fact, so poor that no careful buyer would purchase them. We feel it is not only misleading to buyers, but also detrimental to manufacturers, to have such prices published broadcast.

Yours truly,
(Sgd.) The Liverpool Lint Co.
Mark Street Mills, Netherfield Road North, Liverpool,
May 25, 1910.

